# Repurposing of the ALK Inhibitor Crizotinib for Acute Leukemia and Multiple Myeloma Cells

**DOI:** 10.3390/ph14111126

**Published:** 2021-11-05

**Authors:** Joelle C. Boulos, Mohamed E. M. Saeed, Manik Chatterjee, Yagmur Bülbül, Francesco Crudo, Doris Marko, Markus Munder, Sabine M. Klauck, Thomas Efferth

**Affiliations:** 1 Department of Pharmaceutical Biology, Institute of Pharmaceutical and Biomedical Sciences, Johannes Gutenberg University, Staudinger Weg 5, 55128 Mainz, Germany; joboulos@uni-mainz.de (J.C.B.); saeedm@uni-mainz.de (M.E.M.S.); 2 Translational Oncology, Comprehensive Cancer Center Mainfranken, University Hospital Würzburg, 97078 Würzburg, Germany; Chatterjee_M@ukw.de; 3 Third Department of Medicine (Hematology, Oncology, and Pneumology), University Medical Center of the Johannes Gutenberg University, 55131 Mainz, Germany; Yagmur.Buelbuel@sanofi.com (Y.B.); munder@uni-mainz.de (M.M.); 4 Department of Food Chemistry and Toxicology, University of Vienna, Währinger Str. 38, 1090 Wien, Austria; francesco.crudo@univie.ac.at (F.C.); doris.marko@univie.ac.at (D.M.); 5 Division of Cancer Genome Research, German Cancer Research Center (DKFZ), German Cancer Consortium (DKTK), National Center for Tumor Diseases (NCT), 69120 Heidelberg, Germany; s.klauck@dkfz-heidelberg.de

**Keywords:** acute myeloid leukemia, drug repurposing, multiple myeloma, network pharmacology, transcriptomics, tyrosine kinase inhibitors

## Abstract

Crizotinib was a first generation of ALK tyrosine kinase inhibitor approved for the treatment of *ALK*-positive non-small-cell lung carcinoma (NSCLC) patients. COMPARE and cluster analyses of transcriptomic data of the NCI cell line panel indicated that genes with different cellular functions regulated the sensitivity or resistance of cancer cells to crizotinib. Transcription factor binding motif analyses in gene promoters divulged two transcription factors possibly regulating the expression of these genes, i.e., RXRA and GATA1, which are important for leukemia and erythroid development, respectively. COMPARE analyses also implied that cell lines of various cancer types displayed varying degrees of sensitivity to crizotinib. Unexpectedly, leukemia but not lung cancer cells were the most sensitive cells among the different types of NCI cancer cell lines. Re-examining this result in another panel of cell lines indeed revealed that crizotinib exhibited potent cytotoxicity towards acute myeloid leukemia and multiple myeloma cells. P-glycoprotein-overexpressing CEM/ADR5000 leukemia cells were cross-resistant to crizotinib. NCI-H929 multiple myeloma cells were the most sensitive cells. Hence, we evaluated the mode of action of crizotinib on these cells. Although crizotinib is a TKI, it showed highest correlation rates with DNA topoisomerase II inhibitors and tubulin inhibitors. The altered gene expression profiles after crizotinib treatment predicted several networks, where *TOP2A* and genes related to cell cycle were downregulated. Cell cycle analyses showed that cells incubated with crizotinib for 24 h accumulated in the G_2_M phase. Crizotinib also increased the number of p-H3(Ser10)-positive NCI-H929 cells illustrating crizotinib’s ability to prevent mitotic exit. However, cells accumulated in the sub-G_0_G_1_ fraction with longer incubation periods, indicating apoptosis induction. Additionally, crizotinib disassembled the tubulin network of U2OS cells expressing an α-tubulin-GFP fusion protein, preventing migration of cancer cells. This result was verified by in vitro tubulin polymerization assays. In silico molecular docking also revealed a strong binding affinity of crizotinib to the colchicine and *Vinca* alkaloid binding sites. Taken together, these results demonstrate that crizotinib destabilized microtubules. Additionally, the decatenation assay showed that crizotinib partwise inhibited the catalytic activity of DNA topoisomerase II. In conclusion, crizotinib exerted kinase-independent cytotoxic effects through the dual inhibition of tubulin polymerization and topoisomerase II and might be used to treat not only NSCLC but also multiple myeloma.

## 1. Introduction

Multiple myeloma (MM) is the second most common hematologic malignancy with 32,270 new cases and 12,830 deaths estimated to have occurred in the United States in 2020 [1]. It is characterized by the aberrant proliferation of clonal plasma cells in the bone marrow, which causes hypercalcemia, renal impairment, anemia, lytic bone lesions, and immune compromise [1]. Cytogenetic and molecular abnormalities classify MM into hyperdiploid (HD) and non-hyperdiploid (NHD). NHD is also divided into different subgroups: hypodiploid, pseudodiploid, and tetraploid [2], originating from the doubling of both hypodiploid and pseudodiploid karyotypes [3]. This heterogeneity is one among several factors contributing to the limited efficacy of chemotherapeutic drugs, including doxorubicin, cyclophosphamide, and melphalan [4]. In the past decade, several novel therapeutic strategies have been established to treat MM patients, especially proteasome inhibitors, such as bortezomib [5], immunomodulatory drugs (IMiDs, e.g., lenalidomide), and monoclonal antibodies (e.g., daratumumab, elotuzumab, and isatuximab) [6]. In vitro and in vivo experiments as well as clinical trials in both front-line and relapsed MM patients showed that these therapeutic agents induced cytotoxicity against MM cells in the bone marrow [7]. Despite advances in supportive and systemic treatments, MM is still incurable with a high percentage of relapsed patients due to primary or secondary drug resistance [8]. Thus, there is a high medical need for novel therapies and innovative methodologies are being intensively sought.

The classical process of drug development is time-consuming and cost-intensive. An alternative approach is to find new treatment indications for already approved drugs. This approach is known as “drug repositioning or repurposing”. The main advantage of drug repurposing is that toxicity profiles, pharmacodynamics, as well as pharmacokinetics of a marketed drug are well-known. If a drug repurposing approach is successful, a considerable cut in money expenditures and time can be achieved during preclinical and clinical evaluation of the drug [9].

**Figure 1:**
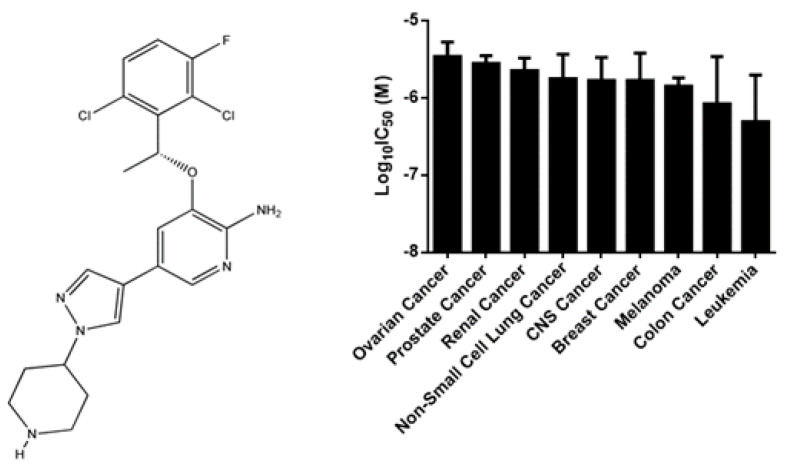
Chemical structure of crizotinib and mean log_10_IC_50_ values ± SD of crizotinib in 59 NCI cancer cell lines.

Crizotinib is a small molecule that inhibits receptor tyrosine kinases (Figure 1). It was first developed as inhibitor of c-MET, but it also exhibited potent inhibition of anaplastic lymphoma kinase (ALK) phosphorylation as well as signal transduction. Translocations affecting the *ALK* gene lead to the expression of oncogenic ALK fusion proteins and increased cancer cell proliferation. Crizotinib inhibits ALK and c-MET phosphorylation in a dose-dependent manner. In vitro and in vivo experiments revealed that crizotinib induced G1S phase cell cycle arrest followed by apoptosis. Crizotinib also inhibited ROS1 receptor tyrosine kinase [10]. Consequently, in August 2011, the US Food and Drug Administration (FDA) approved crizotinib for the treatment of *ALK*-positive non-small-cell lung cancer patients (NSCLC) [11]. Crizotinib also induced cytotoxicity against breast cancer cells [12]. A case report showed that crizotinib was highly effective in a metastatic papillary renal carcinoma [13]. Other case reports revealed that *ALK*-translocated inflammatory myofibroblastic tumors (IMT) [14] and *ALK*-positive anaplastic large cell lymphoma (ALCL) showed encouraging response rates to crizotinib, including completely responding patients [15,16,17]. These clinical data can be taken as indication that the activity of crizotinib is not restricted to NSCLC. However, no studies have been conducted to study the response of acute myeloid leukemia (AML) and MM to crizotinib. Thus, the aim of this study was to investigate the effect of crizotinib in MM and AML cancer cells.

## 2. Results

### 2.1. Cytotoxicity of Crizotinib

**Figure 2:**
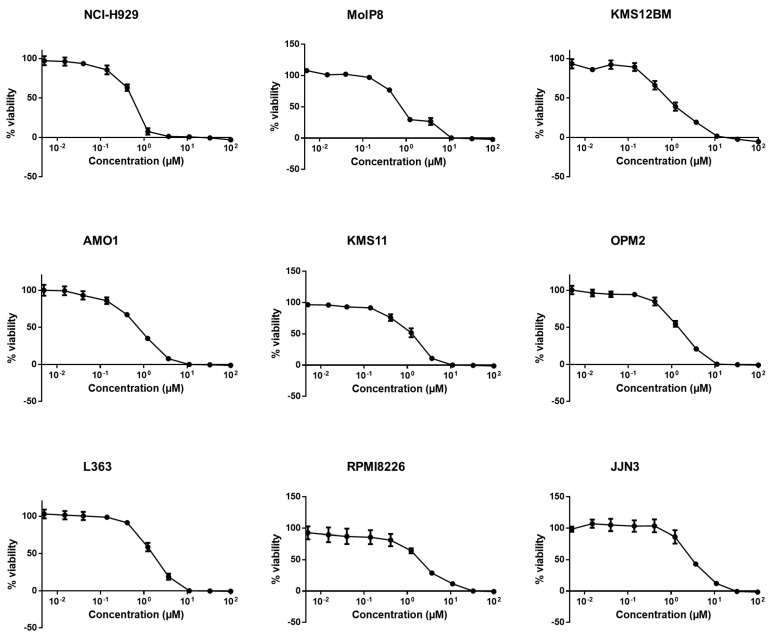
Cytotoxicity of crizotinib in human MM cell lines: NCI-H929, MolP8, KMS12BM, AMO1, KMS11, OPM2, L363, RPMI8226, and JJN3. Each point represents the mean value ± SD of three independent experiments with six replicates each.

Resazurin assays revealed that all tested MM cell lines were sensitive to crizotinib 72 h post-treatment. The most sensitive cell line was NCI-H929 (IC_50_: 0.53 ± 0.04 µM), and the least sensitive cell line was JJN3 (IC_50_: 3.01 ± 0.39 µM) (Figure 2, Table 1).

**Figure 3:**
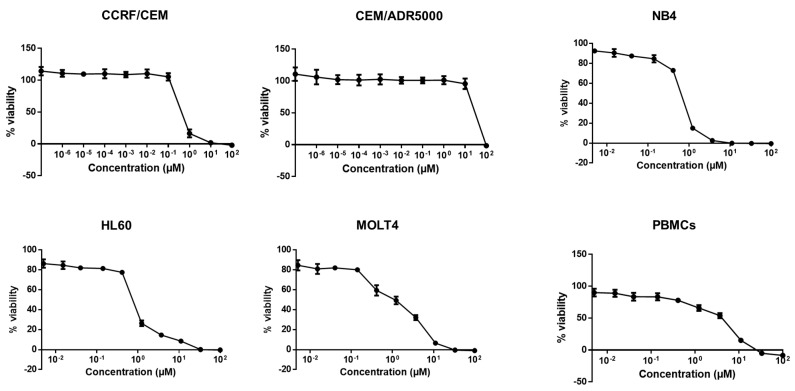
Cytotoxicity of crizotinib in human AML cell lines (CCRF/CEM, CEM/ADR5000, NB4, HL60, and MOLT4) and PBMCs. Each point represents the mean value ± SD of three independent experiments with six replicates each.

Moreover, all leukemia cell lines were responsive to crizotinib too, with CCRF-CEM as the most sensitive (IC_50_: 0.43 ± 0.07 µM) and CEM/ADR5000 as the least sensitive cell line (IC_50_: 29.15 ± 2.59 µM) (Figure 3, Table 1). As CEM/ADR5000 cells are a multidrug-resistant subline of CCRF-CEM cells, CEM/ADR5000 cells were 67.8-fold cross-resistant to crizotinib compared to CCRF-CEM cells. The concentration of crizotinib required to inhibit viability in 50% of PBMCs (4.02 ± 0.49 µM) (Figure 3) was higher than the highest concentration needed to inhibit MM cancer cells, but lower than the concentration needed to inhibit multidrug-resistant CEM/ADR5000 leukemia cells.

**Figure 4:**
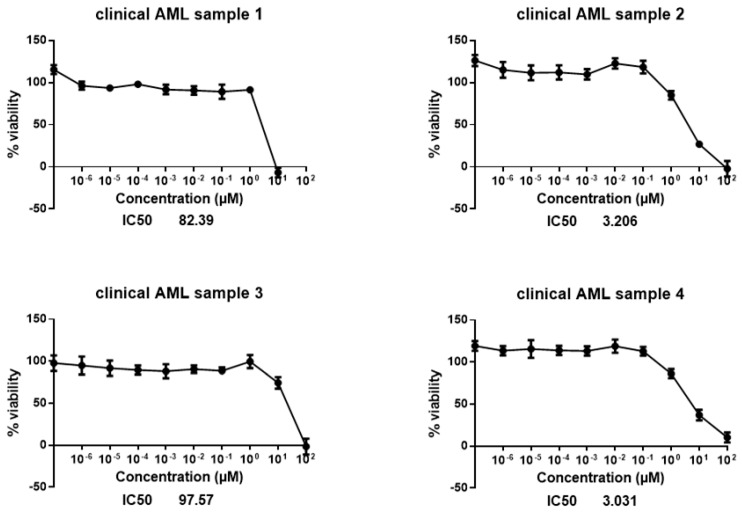
Cytotoxicity of crizotinib in clinical leukemia samples.

To see whether crizotinib is only highly cytotoxic in established cell lines or also in clinical leukemia, we isolated leukemic blasts from blood samples of leukemia patients. The cytotoxicity of crizotinib varied in four clinical leukemia samples with IC50 values ranging from 3.031 µM to 97.57 µM (Figure 4).

### 2.2. Cross-Resistance of Crizotinib to Established Anti-Cancer Drugs

To decipher possible mechanisms of action of crizotinib, we correlated the log_10_IC_50_ values of 59 NCI cancer cell lines towards crizotinib with 87 standard drugs. The cellular responses of 8 out of 9 DNA topoisomerase II inhibitors correlated with crizotinib (88.89%). Moreover, tubulin inhibitors significantly correlated with crizotinib (80%), and four out of five tubulin inhibitors significantly correlated with crizotinib (*R* > 0.252, *p* < 0.029). Similar results were found for alkylating drugs (61.53%), tyrosine kinase inhibitors (57.14%), and antibiotics (50%).

**Figure 5:**
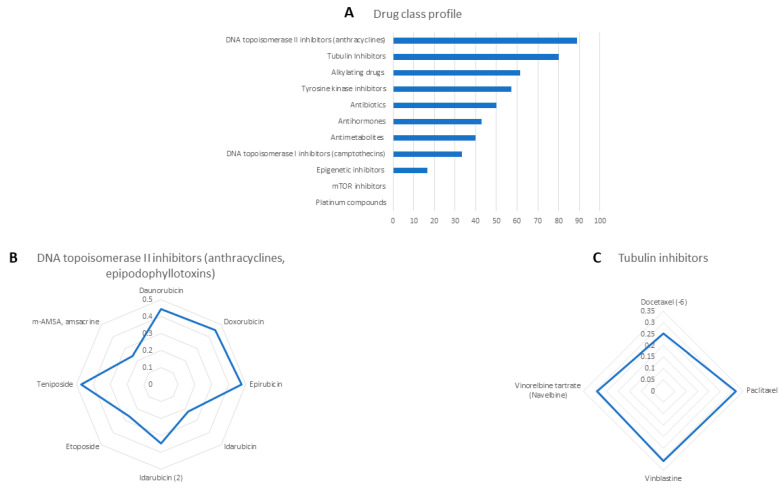
Oncobiograms for crizotinib using the panel of NCI cancer cell lines. (**A**) Percentage of 87 standard drugs whose log_10_IC_50_ values correlated with crizotinib. (**B**) Profile of correlation values of DNA topoisomerase II inhibitors with crizotinib. (**C**) Profile of correlation values of tubulin inhibitors with crizotinib. All the calculations were carried out using Pearson correlation test.

Less stringent associations were obtained with antihormones (42.86%), antimetabolites (40%), DNA topoisomerase I inhibitors (33.33%), and epigenetic inhibitors (16.67%). No associations were obtained with mTOR inhibitors and platinum compounds. These results suggest that crizotinib might inhibit DNA topoisomerase II as well as tubulin (Figure 5).

### 2.3. COMPARE and Hierarchical Cluster Analyses of Transcriptome-Based Expression Profiling of the NCI Tumor Cell Line Panel

**Figure 6:**
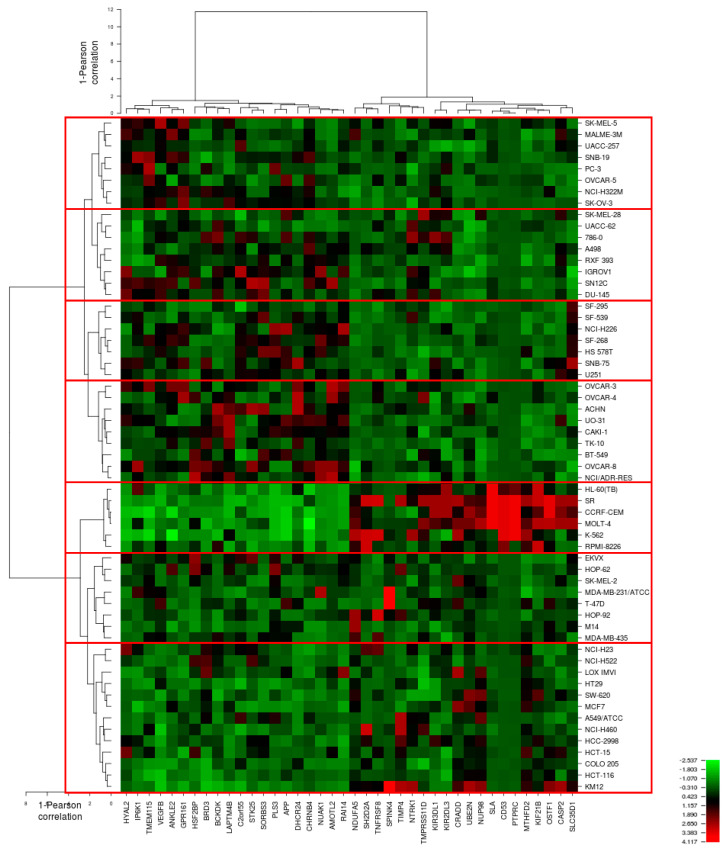
Transcriptomic heat map of crizotinib obtained by hierarchical cluster analysis of 59 cancer cell lines and mRNA expression of genes that directly or inversely correlated with the log_10_IC_50_ values of crizotinib. The red boxes represent the separation of 59 NCI cancer cell lines into seven clusters.

In the present investigation, we performed two different transcriptomic analyses. The first analysis focused on a comparison of gene expression and the correlation of log_10_IC_50_ values of crizotinib in these cell lines (Figure 6, Table 2 and Table 3). In this approach, the influence of molecular architecture of untreated cells on the crizotinib responsiveness was investigated. The second transcriptomic approach focused on altered gene expression profiles after crizotinib treatment and is reported in Section 2.5.

The transcriptome-wide mRNA expressions of the 59 NCI tumor cell lines were mined and correlated to the log_10_IC_50_ values of crizotinib. COMPARE analysis was performed to rank these genes based on direct or inverse correlation of mRNA expression and log_10_IC_50_ values for crizotinib. Forty genes with the best correlation coefficients were identified, 20 genes were directly correlated to the log_10_IC_50_ values of crizotinib, and the other 20 genes were inversely correlated to the log_10_IC_50_ values of crizotinib (Table 2).

Afterwards, hierarchical cluster analysis of the mRNA expression values of the genes of the 59 cancer cell lines was performed to discover whether clusters of cell lines with identical behavior upon exposure to crizotinib could be identified. The dendrogram of hierarchical cluster analysis revealed seven clusters with statistical significance (*p* = 0.011) as shown in the heat map of Figure 6. The majority of cell lines in clusters 1, 3 and 4 were resistant to crizotinib, whereas the majority of cell lines in clusters 5, 6, and 7 were sensitive to crizotinib. Cluster 2 was of a mixed type and contained evenly sensitive and resistant cell lines (Table 3).

The median of log_10_IC_50_ value of crizotinib was taken as cut-off in order to categorize cells of each cluster as sensitive or resistant.

COMPARE and hierarchical cluster analysis revealed that cancer cells of distinct tumor types exhibit varying degrees of sensitivity to crizotinib, with leukemia being the most sensitive tumor type (Figure 1).

### 2.4. Transcription Factor Binding Motif Analysis

**Figure 7:**
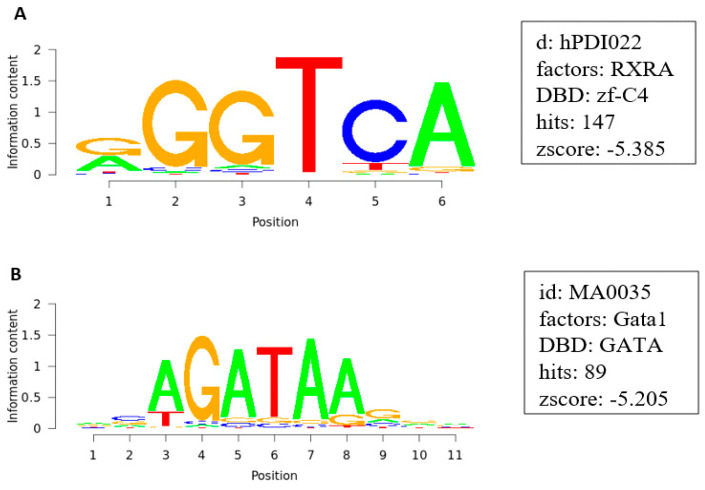
Binding motifs of RXRA (**A**) and GATA1 (**B**).

Motif analyses of 25,000 bases upstream of the promoter region of the genes identified by COMPARE were performed, in order to examine transcription factors possibly regulating the expression of these genes. The analysis revealed the presence of RXRA (147 hits), a transcription factor required for leukemia development. It also revealed the presence of GATA1 (89 hits), a transcription factor important for erythroid development (Figure 7).

### 2.5. Transcriptome-Based Expression Profiling of Crizotinib-Treated NCI-H929 Cells

NCI-H929 cells were treated with the corresponding IC_50_ concentration of crizotinib and subjected to microarray-based mRNA profiling. The genes, which were differentially expressed compared to untreated cells, were analyzed using the Ingenuity Pathway Analysis (IPA) (Qiagen, Hilden, Germany) software.

**Figure 8:**
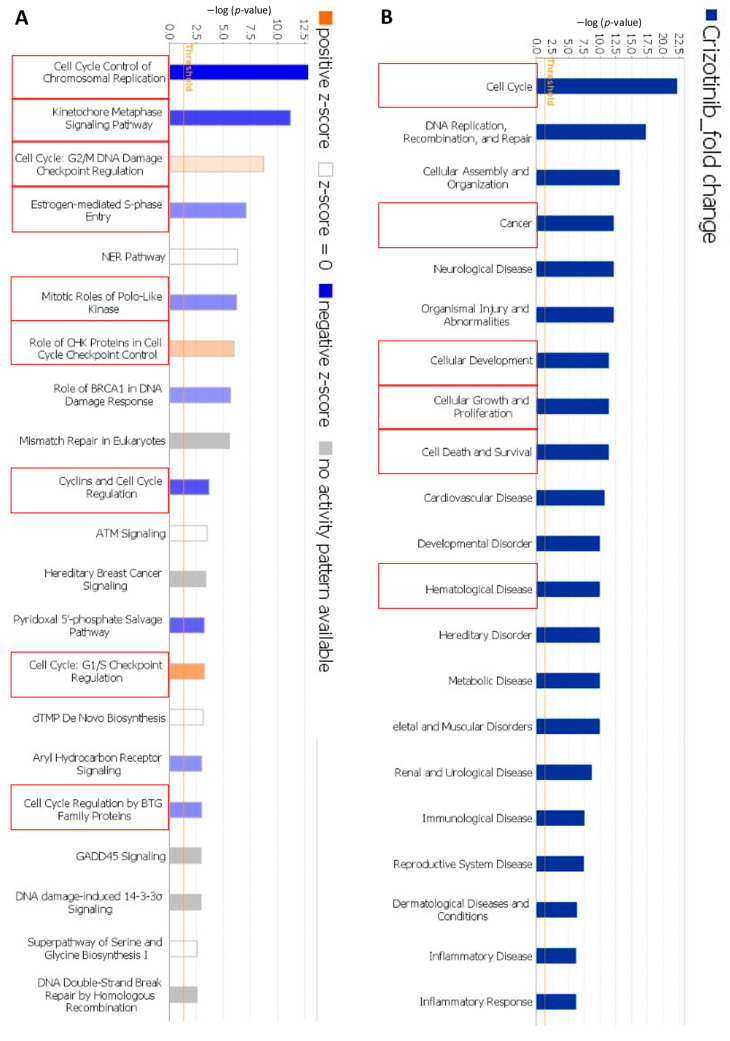
Top functional (**A**) and canonical (**B**) pathways affected by crizotinib as determined by IPA and highlighted by the red boxes.

IPA predicted important cellular function and diseases that could be affected by crizotinib, e.g., cell cycle, cellular growth and proliferation, cellular development, cell death and survival, cancer, and hematological diseases (Figure 8A). Moreover, IPA revealed significant canonical pathways all related to cell cycle regulation (Figure 8B).

**Figure 9:**
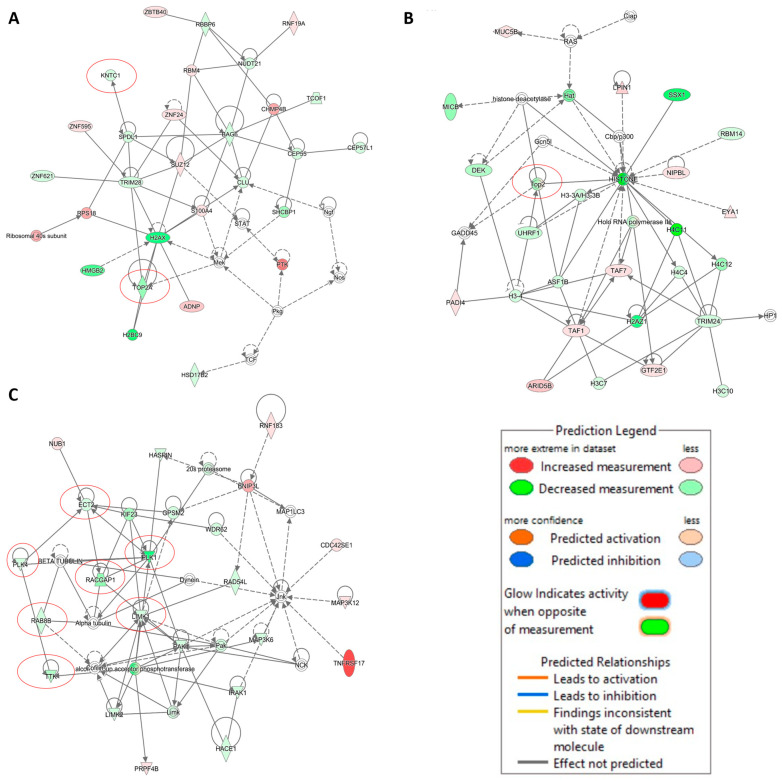
Prediction of functional networks by transcriptome-wide microarray hybridization and IPA of crizotinib-treated NCI-H929 cells. The red circles highlight the downregulation of TOPO2A (network (**A**,**B**)) and genes related to cell cycle, mitosis, and tubulin (network (**C**)).

IPA also predicted a network where both *KNTC1* and *TOP2A* were downregulated (Figure 9, Network A). Network B also revealed that *TOP2* was downregulated (Figure 9, Network B). Genes related to cell cycle, mitosis, and tubulin were also downregulated (Figure 9, Network C). These findings were in line with our hypothesis that crizotinib might inhibit microtubules and DNA topoisomerase 2A.

### 2.6. Cell Cycle Analysis

**Figure 10:**
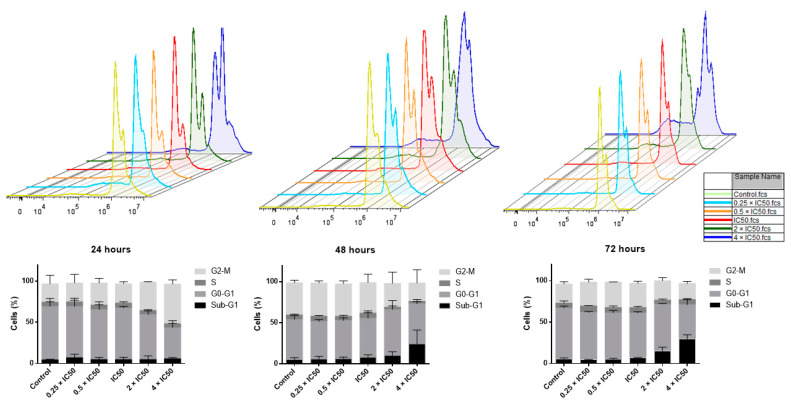
Cell cycle analysis of NCI-H929 cells treated with different crizotinib concentrations for 24, 48, and 72 h. The statistical analysis shows mean values ± SD of three independent experiments.

To investigate cell cycle perturbations by crizotinib, the cell cycle status of NCI-H929 cells was determined following 24, 48, or 72 h incubation with different crizotinib concentrations. Twenty-four hours after treatment, high crizotinib concentrations (2 × IC_50_ and 4 × IC_50_) decreased the fraction of NCI-H929 cells in the G_0_G_1_ phase and increased the fraction of cells in the G_2_M phase (Figure 10). After 48 and 72 h treatment, the fraction of cells in sub G_0_G_1_ phase considerably increased at high concentrations (2 × IC_50_ and 4 × IC_50_), suggesting the induction of apoptosis by crizotinib (Figure 10).

### 2.7. Immunofluorescence of Phospho-Histone-3 (Ser10)

**Figure 11:**
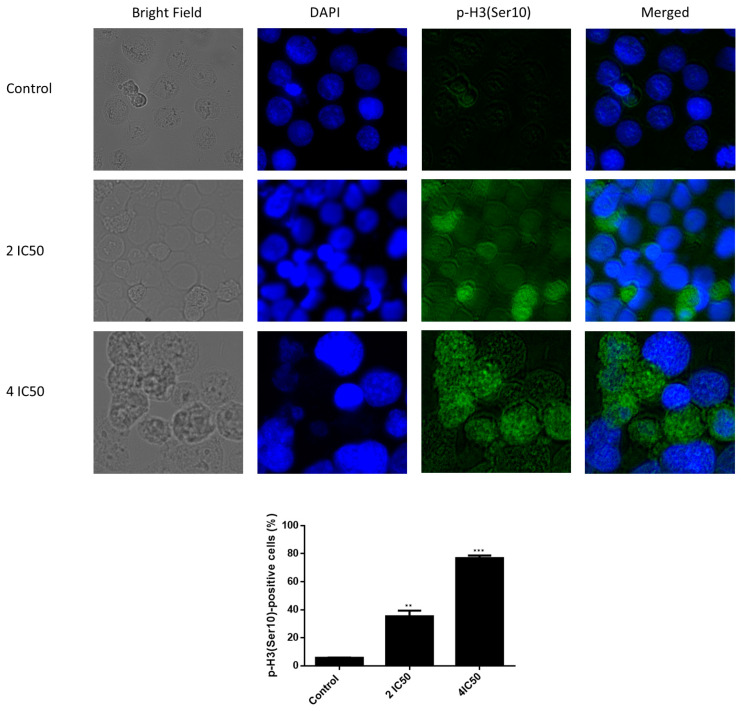
Accumulation of p-H3(Ser10)-positive NCI-H929 cells treated with crizotinib or DMSO (control) for 24 h. NCI-H929 cells were immunostained with anti-phospho-histone H3 (Ser10) clone 3H10, FITC-conjugated antibody (green). Images were merged with DAPI (blue) to represent the nucleus (×40 magnification). ** *p* ≤ 0.01 and *** *p* ≤ 0.001, if compared to untreated control cells.

To verify the accumulation of NCI-H929 cells in the M-phase, the number of mitotic cells upon 24 h treatment with crizotinib was quantified by staining of p-H3(Ser10) and nuclear co-staining. In fact, an obvious increase in the number of p-H3(Ser10)-positive NCI-H929 treated cells was detected (Figure 11). For instance, the number of mitotic cells in NCI-H929 treated with 2 × IC_50_ value of crizotinib (~37%) increased around eight-fold compared to control cells (~5%). This number dose-dependently increased to reach around 80% of p-H3(Ser10)-positive cells with 4 × IC_50_. Therefore, crizotinib induced the accumulation of MM cells in the M-phase.

### 2.8. Effect of Crizotinib on Microtubules

**Figure 12:**
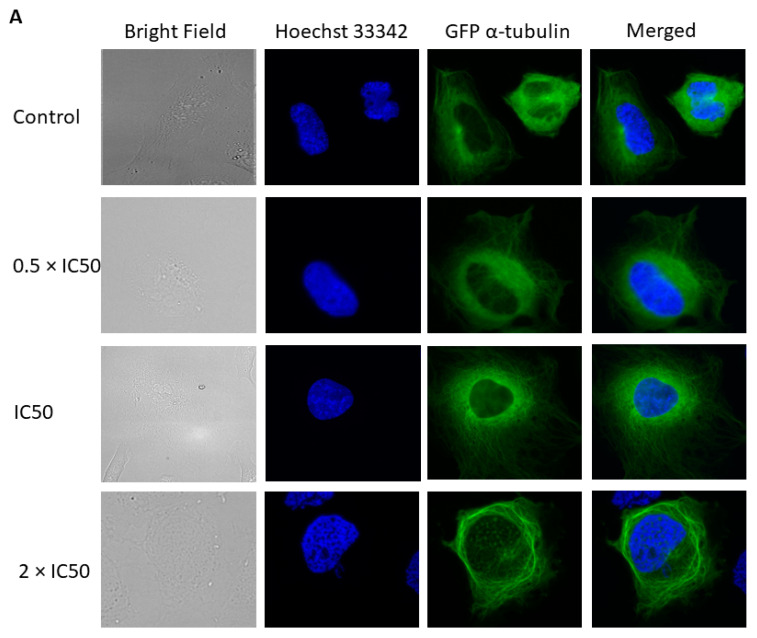

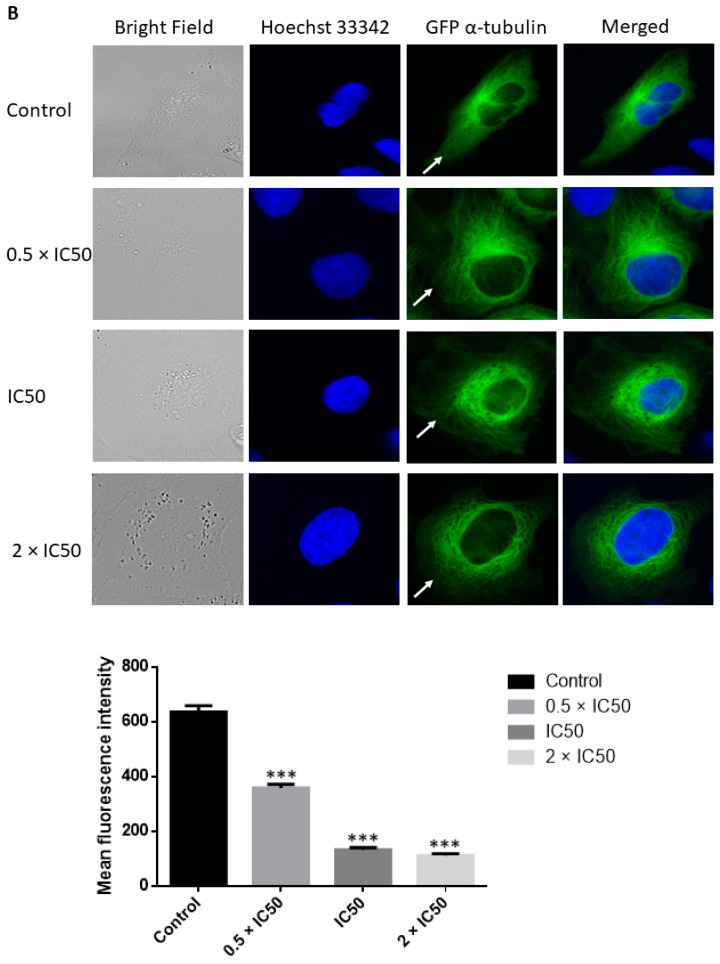
Alteration of the microtubule arrangement in U2OS cells upon treatment with crizotinib. Panel (**A**) reveals micrographs of live cell imaging of U2OS cells 1 h post-incubation on ice with DMSO and various crizotinib concentrations. Panel (**B**) reveals micrographs of live cell imaging of U2OS cells 1 h post-incubation on ice with DMSO and different crizotinib concentrations, followed by 24 h incubation at 37 °C and 5% CO_2_). Images were merged with Hoechst 33342 (blue) to illustrate cell nuclei (×40 magnification). The white arrows represent the microtubule masses at the periphery. The graph shows the mean fluorescence intensity of U2OS cells expressing α-tubulin-GFP after 24 h treatment with DMSO and different crizotinib concentrations. *** *p* ≤ 0.001, if compared to untreated control cells.

To assess the effect of crizotinib on the microtubule network, U2OS cells expressing α-tubulin-GFP fusion protein were treated with different concentrations of crizotinib (0.5 × IC_50_, IC_50_, and 2 × IC_50_) for 24 h. After adding DMSO or the appropriate drug concentrations, cells were placed on ice for 1 h. As shown in Figure 12A, tubulin is disintegrated in control cells, and the degree of disintegration is increasing upon treatment in a dose-dependent manner. After 24 h incubation at 37 °C and 5% CO_2_, the tubulin of control cells polymerized again, clearly noticed by the far distribution of the tubulin through the cytoplasm creating a dense intracellular network in the cytoplasm. By contrast, an abnormal microtubule arrangement was still observed in crizotinib-treated cells. The mass of the microtubules decreased at the periphery and increased concurrently around the nucleus. The brightness as well as the thickness of microtubules at the periphery of treated cells diminished compared to untreated control cells. Together, these results revealed that crizotinib inhibited the microtubule network (Figure 12B).

### 2.9. Effect of Crizotinib on Microtubule Polymerization

**Figure 13:**
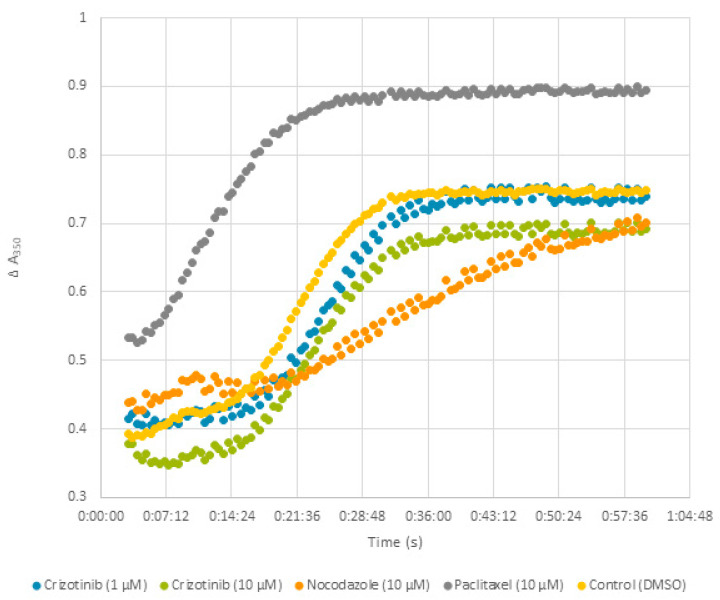
Inhibition of microtubule polymerization by crizotinib. Tubulin was incubated with crizotinib (1 and 10 µM), paclitaxel (10 µM), nocodazole (10 µM), and DMSO at 37 °C. Light scattering was measured every 30 s for 1 h. A shift of the curve to the upper left of DMSO indicated an increase in microtubule polymerization. A shift to the lower right of DMSO reflected a decrease in polymerization.

To investigate the molecular mode of action of crizotinib on microtubule assembly, an in vitro tubulin polymerization assay was performed, where paclitaxel and nocodazole were used as positive controls for microtubule-stabilizing agents (MSAs) and microtubule-destabilizing agents (MDAs), respectively. As anticipated in Figure 13, paclitaxel strongly increased microtubule polymerization, while nocodazole significantly restrained this process. At low crizotinib concentrations, a slight decrease in light scattering was detected at 350 nm, and this decrease became more significant at higher concentrations. Together, these results indicated that crizotinib inhibited tubulin polymerization in a dose-dependent-manner, and it acted similar to MDAs.

### 2.10. Molecular Docking of Crizotinib to Colchicine and Vinca Alkaloids Binding Sites

**Figure 14:**
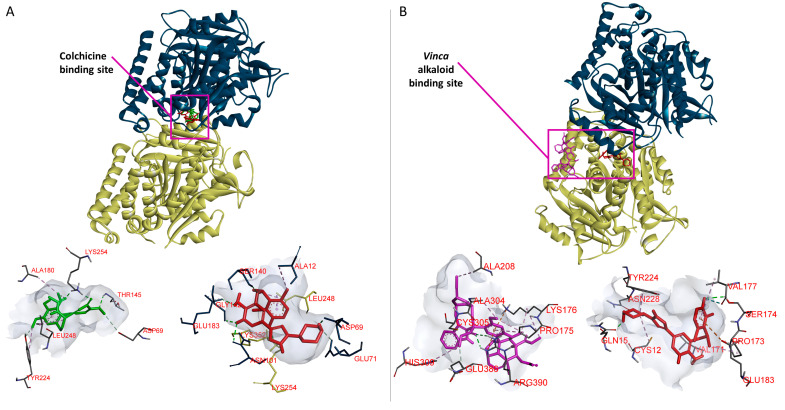
Visualization of the defined molecular docking results. α- (cyanide color) and β- tubulin (creamy white color) are shown in new cartoon format. Crizotinib is represented in red, colchicine in green, and vinorelbine in purple. Panel (**A**) illustrates the result of the in silico defined docking of crizotinib and colchicine binding in the same pocket of the colchicine binding site. Panel (**B**) illustrates the result of the defined docking of crizotinib and vinorelbine binding to distinct pockets of the *Vinca* alkaloids binding site. Each ligand is shown with the interacting amino acids of the corresponding binding site.

Like MDAs, crizotinib inhibited microtubule polymerization. Defined molecular docking was performed to determine the binding affinity of crizotinib to the best studied microtubule destabilizing sites, i.e., the colchicine binding site and the *Vinca* alkaloid binding site. Crizotinib exhibited approximately the same binding affinity to the *Vinca* alkaloid binding site as to the colchicine binding site (Table 4). GLU183, a residue found in both binding sites, was involved in crizotinib binding to the colchicine and the *Vinca* alkaloid binding sites. Crizotinib also exerted a stronger binding affinity to the colchicine binding site and to the *Vinca* alkaloid binding site, if compared to colchicine and vinorelbine, respectively (Table 4). Furthermore, crizotinib bound to the same binding pocket as colchicine in the colchicine binding site (Figure 14A). Crizotinib and colchicine shared three amino acids involved in hydrophobic or H-bond interactions: ASP69, LEU248, and LYS254. However, crizotinib bound to a distinct pocket than vinorelbine in the *Vinca* alkaloid binding site (Figure 14B). The defined molecular docking results confirmed that crizotinib inhibited microtubule polymerization by strongly binding to colchicine and *Vinca* alkaloid binding sites.

### 2.11. Influence of Crizotinib on Human Topoisomerase II Activity

**Figure 15:**
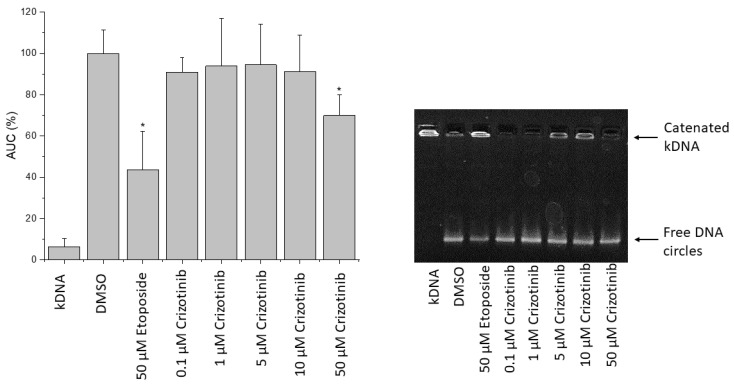
Effect of crizotinib on the catalytic activity of human topoisomerase II. Topoisomerase II (2 U) was incubated at 37 °C for 1 h in absence (DMSO, lane 2) or presence of crizotinib (lanes 4–8) or etoposide (lane 3). Represented are the fluorescence intensities of the decatenated kDNA which were normalized to the control (DMSO) and an illustrative gel. The bars represent the mean value (±) standard deviation of three independent experiments. * *p* ≤ 0.5 if compared to untreated control.

The impact of crizotinib on human topoisomerase II was assessed using the decatenation assay. Catenated kDNA was not able to travel through the agarose gel, as opposed to DNA-minicircles that are released from catenated DNA aggregate by active topoisomerase II. The incubation of kDNA and human topoisomerase II for 1 h in the presence of crizotinib revealed that low concentrations of crizotinib did not significantly decrease topoisomerase II activity. However, a concentration of 50 µM of crizotinib significantly reduced topoisomerase II activity by 30%, if compared to the solvent control (Figure 15). At the same concentration, the positive control etoposide decreased topoisomerase II activity by 58%. All in all, crizotinib inhibited topoisomerase II activity at high concentrations.

## 3. Discussion

Crizotinib was the first generation of ALK tyrosine kinase inhibitors applied for the treatment of *ALK*-positive NSCLC patients [18]. It is an ATP-competitive small-molecule that inhibits RTKs, such as ALK, c-Met, and ROS1, which arrests the cell cycle in the G1/S phase and induces apoptosis [10,19].

The aim of the present study was to investigate the modes of action of crizotinib in more detail. As a first step, the investigation of the NCI tumor cell lines consisting of different tumor types (leukemia, brain cancer, melanoma, carcinoma of the lung, breast, ovary, colon, or prostate) revealed that although crizotinib was originally approved to treat NSCLC, leukemia but not lung cancer cells were the most crizotinib-sensitive cells (Figure 1). Hence, crizotinib might be a new indication for leukemia treatment. As the NCI leukemia cell lines encompass not only leukemia sensu strictu, but also contained one multiple myeloma cell line (RPMI8226), crizotinib may also be repurposed for this tumor entity.

Therefore, we decided to investigate a panel of five leukemia and nine multiple myeloma cell lines independent of the NCI cell line panel in more detail. All these cell lines, except the multidrug-resistant CEM/ADR5000 cells, were sensitive towards crizotinib. This result validated the results we observed with the NCI tumor line panel. Multidrug-resistant CEM/ADR5000 cells overexpressed the ABC-transporter P-glycoprotein, known to expel a large variety of chemically and functionally diverse anticancer drugs out of tumor cells, which ultimately can lead to the failure of chemotherapy in the clinical setting [20]. Our data suggested that P-glycoprotein also recognized crizotinib as substrate and that crizotinib is part of the multidrug resistance phenotype. The concentrations of crizotinib to inhibit normal leukocytes (IC_50_: 4.02 ± 0.49 µM)) were higher than in all leukemia and MM cell lines tested except of multidrug-resistant CEM/ADR5000 cells. This indicates that crizotinib concentrations necessary to kill multidrug-resistant cells could not be achieved without also harming normal cells. We also investigated the in vitro sensitivity of isolated leukemia blasts of four patients towards crizotinib and found that these clinical leukemia cells were also inhibited by crizotinib. However, the degree of sensitivity of these leukemic blasts to crizotinib differed from one patient to another. The blasts with the lowest IC_50_ values were isolated from patients that received two applications of hydroxyurea, which is commonly used as initial treatment to lower leukocyte counts in peripheral blood. The number of samples in this study is low, hence it is not possible to reach a conclusion as to whether the brief in vivo hydroxyurea pretreatment is the only reason behind the low IC_50_ values. Further clinical analyses of crizotinib for leukemia patients are therefore required.

COMPARE analyses were performed with the NCI cell line panel. The expression of genes with different cellular functions was significantly correlated with the responsiveness of these cell lines to crizotinib. These genes encode proteins with various biological activities, as apoptosis (*CRADD*, *CASP2*), angiogenesis (*AMOTL2*, *VEGFB*), transcription (*APP*, *BRD3,* and *HSF2BP*), cytoskeletal organization, cell adhesion and migration (*KIF21B*, *RAI14*, *NUAK1*, *APP*, *STK25*, and *SORBS3*), bones formation and resorption (*PLS3*, *OSTF1*), immune response (*SH2D2A*, *PTPRC*, *SLA*, *TNFRSF8*, *KIR3DL* and *KIR2DL3*), as well as cell development and differentiation (*NTRK1*, *CD53*, and *HYAL2*) (Table 2).

To understand whether the differential expression of these genes could serve as a tool to predict sensitivity or resistance of cancer cells to crizotinib, we performed hierarchical cluster analysis. The mRNA expression profiles of these genes were included in the analysis. The seven clusters obtained from cluster analysis were then correlated with the log_10_IC_50_ values of crizotinib. Indeed, the distribution of cell lines according to their gene expression profiles in the different clusters of the dendrogram was not random, but significantly correlated with cellular responsiveness to crizotinib. Three clusters out of seven consisted mainly of crizotinib-resistant cell lines. Similarly, three clusters out of seven consisted mainly of crizotinib-sensitive cell lines. One cluster contained equally resistant and sensitive cell lines (Figure 6, Table 3). This indicates that this gene expression profiling approach was indeed capable to identify sensitive and resistant tumor cells. Whether this approach is also useful with clinical tumor biopsies deserves further exploration, and gene expression profiling may be used for individualized tumor therapy. If a tumor is tested a priori as crizotinib-sensitive, crizotinib-based chemotherapy may be applied. Provided the individual gene expression profile of a patient’s tumor indicates resistance towards crizotinib, other more promising chemotherapeutics may be applied instead of crizotinib [21].

Although *ALK* expression has also been reported in chronic lymphoblastic leukemia and anaplastic large cell T lymphoma [22,23], it is not common in acute myeloid leukemia and chronic myeloid leukemia or multiple myeloma. Thus, ALK inhibition alone hardly explains the remarkably high sensitivity of our leukemia and multiple myeloma cell lines to crizotinib. Therefore, we postulated that mechanisms other than ALK inhibition contributed to the cytotoxicity of crizotinib. To identify molecular mechanisms other than ALK inhibition, targeted by crizotinib, transcription factor binding motif analysis was performed. We assumed that the genes identified by COMPARE analysis are controlled by common transcription factors. Accordingly, motif analysis of 25,000 bp upstream of the promoter sequence of these genes identified several transcription factors; the top two factors were retinoid X receptor α (RXRA) (Z-score= −5.699) and GATA1 (Z-score= −5.205) (Figure 7). Hence, these two transcription factors might be involved in the regulation of these genes. Retinoid X receptors (RXRs), members of nuclear receptors (NRs), are transcription factors that regulate many cellular processes as cell development, differentiation, proliferation, and apoptosis [24]. In addition of being silent heterodimerization partners of different NRs, RXRα is a target for cancer prevention and therapy because it interacts with its ligands and regulates signaling of related pathways. Retinoid-based differentiation therapy was efficient in treating acute promyelocytic leukemia (APL). 9-Cis-retinoic acid (9-cis-RA) is an RXRα ligand that may be used as treatment in APL and as prevention in skin carcinogenesis. RXRα is an essential feature of the oncogenic complex in APL [25]. Targretin, another RXRα ligand, may serve to treat cutaneous T cell lymphoma [26]. Targretin overcame multidrug resistance in breast cancer and non-small-cell lung cancer [27,28]. COMPARE and hierarchical cluster analyses showed that genes activating apoptosis (*CRADD*, *CASP2*) were resistant to crizotinib, while genes regulating angiogenesis (*AMOTL2*, *VEGFB*) were sensitive to crizotinib. Our findings matched with another study showing that deletion of hepatocyte RXRα in mice downregulated genes associated with angiogenesis, leading to inhibition of angiogenesis, and upregulated genes related to pro-inflammation, adipogenesis, and apoptosis [26]. Retinoid X receptor (RXR) regulates basic functions in myeloid cells, as pro-angiogenic activity, chemokine secretion, phagocytosis, and inflammatory responses. However, its function in tumor-associated myeloid cells is still not well understood [29]. GATA factors control the development of various tissues by repressing or activating transcription. Hence, they play a role in human cancers [30]. GATA1 is an important player in megakaryocyte and erythroid differentiation, thus it regulates various genes involved in survival, differentiation, and proliferation of hematopoietic progenitors [31]. GATA1-related hematological cancers have been previously reported. GATA1 structural mutations were found in Down syndrome patients with megakaryocytic leukemia. Cis-acting regulatory mutations that affect GATA1 expression caused myelofibrosis and acute erythroblastic leukemia in mice. Consequently, imbalanced GATA1 regulation is involved in certain hematological diseases [32]. Overexpression of GATA1 promoted chemotherapy resistance in acute megakaryocytic leukemia and in pancreatic cancer [33,34].

We assumed that crizotinib may target RXRα and GATA1, both of which play a role in hematological malignancies, and based on the fact that targeting RXRα and GATA1 may overcome drug resistance, we thus confirmed our estimations based on classifying the mean log_10_IC_50_ values of crizotinib in 59 NCI cell lines depending on the cancer type of the cells. Therefore, we decided to investigate the effect of crizotinib on AML and MM cancer cells. To the best of our knowledge, this is the first work showing the cytotoxicity and the mechanism of action of crizotinib in myeloma.

To predict potential modes of action of crizotinib, the log_10_IC_50_ values of crizotinib were correlated with the log_10_IC_5_o of 87 standard anticancer drugs. Although crizotinib is a TKI, it showed the highest correlation rates with DNA topoisomerase II inhibitors (88.88%) and tubulin inhibitors (80%). Therefore, crizotinib’s cytotoxicity may be multi-factorial due to its ability to inhibit DNA topoisomerase 2 and tubulin.

Crizotinib exhibited significant cytotoxicity against AML and MM cell lines with remarkable low IC_50_ values. NCI-H929 was the most sensitive cell line. Consequently, we investigated the mode of action of crizotinib in this cell line.

Remarkably, pathway analyses of microarray-based gene expression data revealed several networks, where *TOP2A* and other genes related to cell cycle, mitosis, and tubulin (*KNTC1*, *PLK1*, *PLK4*, *ECT2*, *RACGAP1*, *LIMK1*, *RAB8B*, and *TTK*) were downregulated. This strengthens our assumption that crizotinib inhibits DNA topoisomerase 2 and tubulin.

The cell cycle analysis of NCI-H929 cells treated with different concentrations of crizotinib for 24, 48, and 72 h revealed that cells accumulated in G_2_M phase 24 h post-treatment with crizotinib. Hence, crizotinib may interfere with G_2_M phase mediators. Consequently, we focused on studying the influence of crizotinib on the microtubule cytoskeleton. To examine the molecular mechanisms in charge of the accumulation of cells in the G2/M phase, the number of p-H3(Ser10)-positive NCI-H929 treated cells was determined by immunofluorescence microscopy. Indeed, NCI-H929 cells treated with crizotinib selectively accumulated in the M-phase. Provided that Ser10 phosphorylation emerges in early prophase, it is well-maintained in metaphase, then it starts to reduce in anaphase to completely disappear in telophase beforehand or at the beginning of chromosome decondensation [35]. The increase in the number of p-H3(Ser10)-positive NCI-H929 treated with crizotinib indicates the ability of the latter to prevent mitotic exit in cancer cells. Commonly, the outcome of sustained mitotic arrest is mitotic catastrophe, a process that prevents the proliferation of cells that failed mitosis by using irreversible antiproliferative actions as senescence, necrosis, and apoptosis [36]. This may explain why cells accumulated in the sub G_0_G_1_ phase upon increasing the incubation period to 48 and 72 h.

Microtubules are established by a process that implies the polymerization and depolymerization of α- and β- tubulin heterodimers [37]. Microtubules play an essential role in crucial cellular events, particularly in mitosis, rendering them an interesting target for anti-tumor therapy [38]. Microtubule-targeting agents (MTAs) can be categorized into microtubule-stabilizing agents (MSAs) that enhance polymerization (e.g., taxanes) and microtubule-destabilizing agents (MDAs) that enhance depolymerization (e.g., *Vinca* alkaloids). MTAs alter the dynamic of mitotic spindles initiating the cell cycle check point and leading to G_2_M phase cell cycle arrest [39,40]. Thus, we decided to study the effect of crizotinib on U2OS cells expressing α-tubulin-GFP by analyzing its influence on microtubule cytoskeleton. Indeed, an abnormal microtubule arrangement was observed. The mass of microtubules organized at the periphery decreased and concurrently accumulated around the nucleus. The brightness as well as the thickness of microtubules in treated cells diminished compared to control cells, indicating that crizotinib disassembled the tubulin network. This may be the reason for the cytotoxicity induced by crizotinib. Besides, microtubules play a major role in cell migration [41]. Gene expression profiles revealed that several genes regulating cytoskeletal organization, cell adhesion and migration (*KIF21B*, *RAI14*, *NUAK1*, *APP*, *STK25*, and *SORBS3*) were deregulated. Furthermore, an in vitro tubulin polymerization assay revealed that crizotinib affected microtubule dynamics by depolymerizing microtubules in a comparable manner as MDAs. Hence, crizotinib’s influence on the tubulin network might emphasize its potential to inhibit migration of cancer cells. The doses of crizotinib required in the cell free tubulin polymerization assay were higher than those needed for the in vitro cellular immunofluorescence studies. In fact, tubulin polymerization assay lacks other proteins (e.g., microtubule associated proteins) that are partly responsible for the stabilization or destabilization of microtubules, rendering microtubules considerably dynamic. The microtubules in the tubulin polymerization assay are barely dynamic. Therefore, they may falsify the real intracellular concentration required to destabilize microtubules. Molecular docking revealed that crizotinib displayed the lowest binding energy to the colchicine and *Vinca* alkaloid binding sites with approximately similar binding energies. Given that GLU183 is a common interacting amino acid of crizotinib to the colchicine as well as *Vinca* alkaloid binding sites, it could be that both binding sites overlap and that the colchicine and *Vinca* alkaloid binding sites are analogous sites but at distinct interface subunits [42]. Crizotinib highly bound to the colchicine and *Vinca* alkaloid binding sites, henceforth, crizotinib’s molecular actions are expected to be similar to colchicine and *Vinca* alkaloids. Together, these results indicate that crizotinib destabilizes microtubules similar to MDAs.

Furthermore, being a microtubule destabilizer, the decatenation assay showed that crizotinib inhibited the catalytic activity of topoisomerase II. Topoisomerase II plays a crucial role in mitosis: (i) it generates transient DNA double-strand breaks, (ii) it changes DNA topology, (iii) it controls decatenation check points, and (iv) it regulates the segregation of sister chromatids [43]. All these functions make it a major target in cancer treatment. Topoisomerase II inhibitors and *Vinca* alkaloids were successfully applied as drug cocktails in several cancer treatments. For instance, the combination of doxorubicin, vinblastine, dacarbazine, and bleomycin was highly effective in Hodgkin lymphoma treatment. Additionally, the combination of doxorubicin, vincristine, dexamethasone, and cyclophosphamide was also efficient in acute lymphoblastic leukemia [44,45]. However, the combination of etoposide and colchicine eliminated the effect of each other [46]. Another drawback of these combination therapies is the development of resistance. To overcome the disadvantages of combination therapies, multitarget agents are being sought [47]. Crizotinib, a dual inhibitor that targets microtubules and topoisomerase II, represents an attractive approach for MM treatment, especially because the field of dual microtubule/topoisomerase II inhibitors is still undermined.

## 4. Materials and Methods

### 4.1. Cell Lines and Patient Samples

The Developmental Therapeutics Program of the NCI (Bethesda, MD, USA) established a panel of 59 human tumor cell lines consisting of ovarian, prostate, renal, non-small cell lung, central nervous system, breast, melanoma, colon, and leukemia cancer cells [48]. These cells were treated with crizotinib and the cytotoxicity was evaluated using the sulforhodamine B assay [49] (Figure 1).

Drug-sensitive CCRF-CEM and multidrug-resistant P-glycoprotein-overexpressing CEM/ADR5000 leukemia cells were kindly provided by Dr. Axel Sauerbrey (Children’s Hospital, University of Jena, Jena, Germany).

NB4 leukemia cells were provided by Dr. Gabriele Greve (Medical Center, University of Freiburg, Breisgau, Germany). HL60 leukemia cells were provided by Dr. Andreas Schwarting and Dr. Julia Weinmann-Menke (Medical Center, University Mainz, Mainz, Germany).

The MM cell lines KMS11, KMS12BM, NCI-H929, MolP8, JJN3, OPM2, AMO1, and L363 were provided by Dr. Ellen Leich and Manik Chatterjee, University of Würzburg, Würzburg, Germany. RPMI8226 cells were purchased from the American Type Cell Culture Collection (ATCC^®^ CCL-155™, Manassas, VA, USA). Cells were grown in RPMI 1640 (Life Technologies, Darmstadt, Germany) supplemented with 10% FBS (Life Technologies, Carlsbad, CA, USA) and 1% penicillin (1000 U/mL)/streptomycin (100 μg/mL) (Life Technologies, Carlsbad, CA, USA). Cells were incubated at 37 °C in a 5% CO_2_ incubator.

Fresh blood samples were collected from patients at the Department of Hematology, Oncology and Pneumology (University Medical Center of the Johannes Gutenberg University, Mainz, Germany) and poured in plastic Monovette EDTA tubes. Blood samples were investigated with written informed consent of the patients and with the approval of the ethic committee: Ethikkommission der Landesärztekammer Rheinland-Pfalz, approval Code: 837.1 I 9.1 0 (7 128). Table 5 illustrates the characteristics of the patients enrolled in this study.

Human peripheral blood mononuclear cells (PBMCs) of a healthy donor were isolated using Histopaque^®^ (Sigma–Aldrich, Taufkirchen, Germany). Briefly, 3 mL blood were carefully layered over Histopaque^®^ and centrifuged at 400× *g* for 30 min at 4 °C. Afterwards, the layer containing PBMCs was isolated, washed with PBS, and centrifuged trice at 250× *g* for 10 min. The resulting pellet was re-suspended in 10 mL Panserin 413 growth medium (PAN-Biotech, Aidenbach, Germany), supplemented with 2% phytohemagglutinin M (PHA-M, Life Technologies, Darmstadt, Germany).

Human osteosarcoma cancer cells U2OS transfected with α-tubulin-GFP construct were grown in Dulbecco’s modified Eagle medium (DMEM) (Life Technologies, Darmstadt, Germany) supplemented with 10% FBS (Life Technologies) and 1% penicillin (1000 U/mL)/streptomycin (100 μg/mL) (Life Technologies). Cells were incubated at 37 °C in a 5% CO_2_ incubator. These cells were regularly treated with 250 µg/mL geneticin to endure α-tubulin expression.

### 4.2. Cytotoxicity Assay

The sensitivity of leukemia and MM cells to crizotinib was determined using the resazurin reduction assay, as described previously [50]. A total of 10^4^ cells/well were plated in a flat bottom 96-well plate. Cells were immediately treated with crizotinib (Sigma–Aldrich) in a range of 10 different concentrations, which were each three-fold apart starting with 100 µM to 0.003 µM for MM, HL60 and NB-4 cell lines as well as PBMCs and each 10-fold apart starting with 100 µM to 10^−7^ µM for drug-sensitive CCRF-CEM, multidrug-resistant CEM/ADR5000 cell lines, and leukemia cancer cells isolated from leukemia patients. After 72 h of incubation at 37 °C and 5% CO_2_, 20 μL of resazurin solution (0.01% *w*/*v*; Sigma–Aldrich) were added to each well, then the plates were incubated at 37 °C and 5% CO_2_. Four hours later, the fluorescence of resorufin generated from the reduction of resazurin by viable cells was measured at 544 nm excitation wavelength and 590 nm emission wavelength using an Infinite M2000 Pro plate reader (Tecan, Crailsheim, Germany). Cell viability was plotted against the concentration of crizotinib and the IC_50_ values were determined from three independent experiments with six replicates each using the GraphPad Prism 5 software (GraphPad Software, San Diego, CA, USA) [51].

### 4.3. COMPARE and Hierarchical Cluster Analysis

The NCI (http://dtp.nci.nih.gov (accessed on 20 September 2019) developed a web-based algorithm known as COMPARE analysis, in order to correlate transcriptome-wide mRNA expressions of 59 cancer cell lines to investigational and established anti-cancer agents in terms of their log_10_IC_50_ values. COMPARE analysis is based on ranking Pearson’s correlation coefficient (*R*-value) [52]. Standard and reverse COMPARE analyses were performed to identify genes associated with resistance (positive *R*-values) as well as sensitivity (negative *R*-values) towards crizotinib.

Hierarchical cluster analysis (WARD method) was performed, using CIMMINER program (https://discover.nci.nih.gov/cimminer/ (accessed on 27 September 2019) in order to cluster the mRNA expression of genes in 59 tumor cell lines identified by COMPARE analysis. A two-dimensional dendrogram-based cluster image map (“heat map”) was obtained by integrating different objects based on how close their characters were.

The chi square (χ^2^) test was carried out and the median of crizotinib’s log_10_IC_50_ values was taken as cut-off to examine the linear dependency between resistant and sensitive cell lines [53].

### 4.4. Transcription Factor Promoter Binding Motif Analysis

Gene promoter analyses for transcription factor binding motifs were carried out using Galaxy/Cistrome software (http://cistrome.org/ap/ (accessed on 2 October 2019). Sequences of 40 genes related to cellular responses to crizotinib were converted to BED format by Table Browser in UCSC Genome Browser (https://genome.ucsc.edu/ (accessed on 2 October 2019). Screening of 25 kb upstream regions for all transcription factor-binding motifs was performed on the Cistrome analysis platform using the SeqPos tool [54].

### 4.5. Gene Expression Profiles

Total RNA was extracted from NCI-H929 cells 24 h post-treatment with the corresponding IC_50_ value of crizotinib or with DMSO as a negative control using the RNeasy Kit from Qiagen (Hilden, Germany). Duplicate samples were subjected to microarray hybridization for gene expression profiling using Affymetrix GeneChips^®^ (Affymetrix, Santa Clara, CA, USA) with human Clariom S assays as described previously [55,56,57] at the Genomics and Proteomics Core Facility of the German Cancer Research Center (DKFZ, Heidelberg, Germany).

### 4.6. Cell Cycle Analysis

Cell cycle analysis was performed using propidium iodide (PI) staining as previously described [58]. Briefly, cells were incubated with five different concentrations of crizotinib (0.25 × IC_50_, 0.5 × IC_50_, 1 × IC_50_, 2 × IC_50_, or 4 × IC_50_) or media alone for 24, 48, and 72 h at 37 °C/5% CO_2_. Cells were then harvested and fixed in 80% ethanol at −20 °C. Cells were incubated in PI staining solution (Thermo Fisher Scientific, Dreieich, Germany) for 15 min at 4 °C and read on an Accouri C6 flow cytometer (Becton-Dickinson, Heidelberg, Germany). Total DNA content was measured on FL2-A [59,60].

### 4.7. Fluorescence Microscopy of p-H3(Ser10)

NCI-H929 cells were incubated with two different concentrations of crizotinib (2 × IC_50_ or 4 × IC_50_) or DMSO alone for 24 h at 37 °C/5% CO_2_. Cells were then harvested, washed with TBST (1% TBS + 0.1% Tween 20), and cytospinned on coverslips (Thermo Fisher Scientific, Dreieich, Germany) at 1000 rpm for 5 min. Afterwards, cells were fixed with 4% paraformaldehyde for 15 min at room temperature, washed three times with TBST, and permeabilized with 1% Triton X-100 in TBST at room temperature for 10 min. After permeabilization, cells were washed again three times with TBST. Then, blocking buffer (1% BSA + 10% FBS in TBST) was added to the cells. After 1 h, the blocking buffer was aspirated and 4 µg/mL of the primary antibody anti-phospho-histone H3 (Ser10) clone 3H10, FITC Conjugate (Merck, Darmstadt, Germany) was applied to the slides that were kept at room temperature in a humidified chamber. After 1 h of staining, the slides were washed with TBST trice and 1 µg/mL of 4′,6-diamidino-2-phenylindole (DAPI) (Sigma–Aldrich, Darmstadt, Germany) was applied to each slide for 5 min to stain cell nuclei. Subsequently, slides were washed with TBST trice. Finally, cells were coated with Fluoromount-G^®^ (SouthernBiotech, Birmingham, AL, USA) and examined using a AF7000 widefield fluorescence microscope (Leica Microsystems, Wetzlar, Germany). FITC was detected at 470 nm excitation and 525 nm emission. DAPI was detected at 470 nm excitation and 447 nm emission. Images were analyzed using Fiji ImageJ software (National Institutes of Health, Bethesda, MD, USA) [61].

### 4.8. Fluorescence Microscopy of the Microtubule Cytoskeleton

Human osteosarcoma cancer cells U2OS transfected with α-tubulin-GFP construct were seeded in two µ- Slide 8 Well (10^5^ cells/well) (ibidi, Gräfelfing, Germany) each. Cells were kept overnight in the incubator at 37 °C and 5% CO_2_, then they were treated with different concentrations of crizotinib (0.5 × IC50, IC50, 2 × IC50) or DMSO (negative control) and placed for 1 h on ice. One of the µ-Slide 8 well was stained directly after incubation on ice and the other µ-Slide 8 well was incubated at 37 °C and 5% CO_2_ and stained after 24 h. Cells were washed with PBS and then stained with Hoechst 33342 Nuclear Stain (BioVision, Wiesbaden, Germany) at room temperature in the dark for 30 min. Subsequently, cells were washed with PBS to remove excessive Hoechst stain and mounted with Fluoromount-G^®^. A AF7000 widefield fluorescence microscope (Leica Microsystems, Wetzlar, Germany) was used to perform the imaging. GFP was detected at 470 nm excitation and 525 nm emission. Hoechst stain was detected at 470 nm excitation and 447 nm emission. Images were analyzed using Fiji ImageJ software [61].

### 4.9. Tubulin Polymerization Assay

Crizotinib was tested using the In Vitro Tubulin Polymerization Assay Kit (Merck, Darmstadt, Germany) following the manufacturer’s instructions. The analyses were accomplished using a FlexStation 3 Multi-Mode Microplate Reader (Molecular devices, San Jose, CA, USA) The readings were obtained by measuring the turbidity variation (light scattering) every 30 s for 1 h (121 measurements totally) at 350 nm.

### 4.10. Molecular Docking of Tubulin

The 3-D structures of crizotinib, vinorelbine, docetaxel, and colchicine were downloaded from PubChem (NCBI, Bethesda, MD, USA) [62] as standard data files. The Protein Data Bank (http://www.rcsb.org/ (accessed on 30 June 2021)) was used to download the crystal structure of tubulin [63] as PDB file (PDB code: 5N5N). Molecular docking was performed using AutoDock 4.2.6 (The Scripps Research Institute, San Diego, CA, USA) to determine the in silico binding of crizotinib, vinorelbine, and colchicine to β and α tubulin [64]. Ligands and protein files were turned into Protein Data Bank Partial Charge and Atom Type (PDBQT) files using AutoDockTools 1.5.6. (The scripps Research Institute, La Jolla, CA, USA) A grid box was established to cover the whole protein. The AutoDock build-in Lamarckian algorithm was applied with 250 runs and 25,000,000 evaluations each. The results were acquired from the RMSD cluster analyses of AutoDock. AutoDockTools identified the interacting amino acids. The visualizations were created using BIOVIA Discovery Studio Visualizer (https://discover.3ds.com/ (accessed on 12 July 2021).

### 4.11. Decatenation Assay

To detect the catalytic activity of topoisomerase II, a decatenation assay using catenated kinetoplast DNA (kDNA) was performed. kDNA is a massive network of interlocked DNA minicircles (mainly 2.5 kb) that could be freed by topoisomerase II. The total reaction volume was fixed to 20 µL. In brief, assay buffer (50 mM Tris-HCl; pH 8; 150 mM NaCl; 10 mM MgCl_2_; 2 mM ATP and 0.5 mM DTT) containing kDNA (200 ng) (TopoGen Inc., Buena Vista, CO, USA) and the tested drugs were added to 2 U of human topoisomerase II. After 1 h of incubation at 37 °C, the reaction was ended by adding the stop buffer (TopoGEN Inc.; 5% Sarkosyl, 0.125% bromphenolblue, 25% glycerol). The reaction mixtures were run on a 1% *w*/*v* agarose gels with Tris-acetate/EDTA (TAE) buffer at 4.5 V/cm. Later the gel was placed in 10 mg/L of ethidium bromide solution for 20 min to stain it. The fluorescence intensities of the bands, at the bottom of the gel, representing the decatenated kDNA were used for quantification. Fluorescence signals were detected with Image Quant LAS-4000 (GE Healthcare Life Sciences, Buckinghamshire, UK). Fujifilm Image Gauge software (Fujifilm Medical Systems USA, Inc., Lexington, MA USA) was used to quantify decatenated DNA. Fluorescence intensities were normalized to the solvent control (DMSO; 100%).

## 5. Conclusions

In conclusion, COMPARE analysis identified genes that contribute to the sensitivity or resistance of 59 NCI cancer cell lines towards crizotinib. These genes were regulated by RXRα and GATA1, which are involved in hematological malignancies. Crizotinib showed potent cytotoxicity against all MM and AML cell lines tested. Cell cycle analysis revealed that cells accumulated in the G_2_M phase if incubated for 24 h with crizotinib. However, they accumulated in sub G_0_/G_1_ with longer incubation periods. Crizotinib exhibited a strong inhibitory effect on the tubulin network, restraining migration of cancer cells and leading to mitotic catastrophe. In vitro tubulin polymerization assay and molecular docking showed that crizotinib depolymerized microtubules bound to the colchicine and *Vinca* alkaloid binding sites; henceforth, crizotinib’s molecular actions were expected to be similar to colchicine and *Vinca* alkaloids. Decatenation assay revealed that crizotinib decreased the catalytic activity of topoisomerase IIα. Together, these results indicate that unlike monotargeted therapies known to be monospecific, crizotinib could be a potent chemotherapeutic drug that exhibits kinase-independent cytotoxic effects in MM through the dual inhibition of tubulin polymerization and topoisomerase II.

## Figures and Tables

**Table 1:** IC_50_ values of crizotinib in human MM cells, AML cells and PBMCs.

Cell Type	Cell Line	IC_50_ (µM)
Multiple myeloma	NCI-H929	0.53 ± 0.04
	MolP8	0.77 ± 0.03
	KMS12BM	0.80 ± 0.18
	AMO1	1.21 ± 2.02
	KMS11	1.40 ± 0.40
	OPM2	1.46 ± 0.21
	L363	1.71 ± 0.40
	RPMI8226	1.91 ± 0.18
	JJN3	3.07 ± 0.39
Leukemia	CCRF-CEM	0.43 ± 0.07
	NB4	0.63 ± 0.01
	HL60	0.74 ± 0.02
	MolT4	1.18 ± 0.46
	CEM/ADR5000	29.15 ± 2.59
Normal leukocytes	PBMCs	4.02 ± 0.49

**Table 2:** Correlation of mRNA expression of genes in 59 NCI cancer cell lines with log_10_IC_50_ values of crizotinib.

COMPARE Coefficient	Symbol	Name	Function
0.5363	*TIMP4*	TIMP metallopeptidase inhibitor 4	Inhibitor of matrix metalloproteinases
0.561	*CRADD*	CASP2 and RIPK1 domain containing adaptor with death domain	Apoptosis
0.559	*NTRK1*	Neurotrophic tyrosine kinase, receptor, type 1	Cell differentiation
0.549	*KIF21B*	Kinesin family member 21B	Regulation of microtubule dynamics
0.541	*CD53*	CD53 cell surface molecule	Cell development, activation, growth and motility
0.541	*UBE2N*	Ubiquitin-conjugating enzyme E2N (UBC13 homologue, yeast)	DNA post-replication repair
0.541	*NDUFA5*	NADH dehydrogenase (ubiquinone) 1α subcomplex, 5, 13 kDa	Mitochondrial respiratory chain
0.536	*SH2D2A*	SH2 domain containing 2A	Control of T-cell activation
0.534	*NUP98*	Nucleoporin, 98 kDa	Transport of macromolecules between nucleus and cytoplasm
0.533	*OSTF1*	Osteoclast stimulating factor 1	Induction of osteoclast formation and bone resorption
0.521	*PTPRC*	Protein tyrosine phosphatase, receptor type, C	T-cell activation
0.516	*MTHFD2*	Methylenetetrahydrofolate dehydrogenase (NADP^+^ dependent) 2, methenyl tetrahydrofolate cyclohydrolase	Nuclear-encoded mitochondrial bifunctional enzyme
0.509	*SLA*	SRC-like-adaptor	Negative regulation T-cell receptor (TCR) signaling
0.509	*TNFRSF8*	Tumor necrosis factor receptor superfamily, member 8	Cellular growth and transformation of activated lymphoblasts
0.509	*KIR3DL1*	Killer cell immunoglobulin-like receptor, three domains, long cytoplasmic tail	Inhibition of NK cells activity
0.505	*TMPRSS11D*	Transmembrane protease, serine 11D	Host defense system on the mucous membrane
0.504	*CASP2*	Caspase 2, apoptosis-related cysteine peptidase	Apoptosis
0.504	*KIR2DL3*	Killer cell immunoglobulin-like receptor, two domains, long cytoplasmic tail, 3	Inhibition of NK cells activity
0.503	*SPINK4*	Serine peptidase inhibitor, Kazal type 4	Serine peptidase inhibitor
0.501	*SLC35D1*	Solute carrier family 35 (UDP-glucuronic acid/UDP-N-acetyl galactosamine dual transporter), member D1	Transport of UDP-glucuronic acid (UDP-GlcA) and UDP-N-acetyl galactosamine (UDP-GalNAc) from the cytoplasm into the endoplasmic reticulum
−0.49	*AMOTL2*	Angiomotin like 2	Angiogenesis inhibitor
−0.447	*BCKDK*	Branched chain keto acid dehydrogenase kinase	Regulator of valine, leucine, and isoleucine catabolic pathways
−0.438	*IP6K1*	Inositol hexakisphosphate kinase 1	Member of the inositol phosphokinase family
−0.432	*DHCR24*	24-Dehydrocholesterol reductase	Protection from oxidative stress
−0.432	*ANKLE2*	Ankyrin repeat and LEM domain containing 2	Mitotic regulator
−0.428	*GPR161*	G protein-coupled receptor 161	Potential drug target for triple-negative breast cancer
−0.428	*PLS3*	Plastin 3	Bone development
−0.426	*C2orf55*	Chromosome 2 open reading frame 55	Unknown
−0.425	*RAI14*	Retinoic acid induced 14	Actin regulation
−0.423	*HYAL2*	Hyaluronoglucosaminidase 2	Cell proliferation, migration and differentiation
−0.42	*NUAK1*	NUAK family, SNF1-like kinase, 1	Cell adhesion, cell ploidy and senescence, cell proliferation and tumor progression
−0.418	*APP*	Amyloid β (A4) precursor protein	Involved in cell mobility and transcription regulation
−0.415	*STK25*	Serine/threonine kinase 25	Protein transport, cell adhesion, and cell migration
−0.415	*TMEM115*	Transmembrane protein 115	Retrograde protein transport from Golgi to endoplasmic reticulum
−0.415	*VEGFB*	Vascular endothelial growth factor B	Blood vessels formation
−0.414	*LAPTM4B*	Lysosomal protein transmembrane 4β	Inhibition of EGFR degradation
−0.41	*CHRNB4*	Cholinergic receptor, nicotinic, β4	Opening of an ion-conducting channel across the plasma membrane
−0.41	*BRD3*	Bromodomain containing 3	Transcription
−0.408	*HSF2BP*	Heat shock transcription factor 2 binding protein	Inhibition BNC1 transcriptional activity during spermatogenesis
−0.408	*SORBS3*	Sorbin and SH3 domain containing 3	Cytoskeletal organization, cell adhesion, migration, signaling, gene expression

**Table 3:** Separation of 59 cancer cell lines into clusters (see Figure 6).

Category	Partition (log_10_IC_50_)	Cluster 1	Cluster 2	Cluster 3	Cluster 4	Cluster 5	Cluster 6	Cluster 7
Sensitive	≤−5.771 M	3	4	3	0	5	5	10
Resistant	>−5.771 M	5	4	4	9	1	3	3

χ^2^ test: *p* = 0.011015.

**Table 4:** Results of defined molecular docking. The lowest binding energy and the predicted inhibitory constant pKi are provided for each compound. The amino acids involved in ligand interactions are also listed. The amino acids in bold represent those involved in hydrogen bonds (H-bonds).

Tubulin Binding Sites	Compounds	Lowest Binding Energy (Kcal/mol)	pKi (nM)	Amino Acids Involved in Ligand Interaction
Colchicine binding site	Colchicine	−7.25	4.83 × 10^3^	**LYS254**, **THR145**, **ASP69**, **TYR224**, ALA180, LEU248
	Crizotinib	−9.62	88.6	**GLU183**, **GLY142**, **SER140**, **ASP69**, **GLU71**, **LYS254**, **ASN101**, **LYS352**, ALA12, LEU248,
*Vinca* alkaloid binding site	Vinorelbine	−6.84	9.85 × 10^3^	**GLU386**, **ARG390**, ALA208, ALA304, LYS176, PRO175, CYS305, HIS309
	Crizotinib	−9.24	177.73	**ASN228**, **GLN15**, **VAL171**, **SER174**, PRO173, GLU183, TYR224, CYS12, VAL177

**Table 5:** Characteristics of the patients donating their blood samples for investigational studies.

Sample ID	Primary Diagnosis	Cytogenetics	Molecular Genetics	Leukocytosis at Diagnosis	Treatment Received
Sample 1	AML FAB M4	46 XY	FLT3-ITDhigh	78.000/ul	Untreated
Sample 2	Highly proliferative AML FAB M4Eo	46 XY, CBFB-MYH11 = Inv (16)	FLT3-TKD Mutation	140.000/ul	Two applications of hydroxyurea
Sample 3	Common B-ALL (B-acute lymphoplastic leukemia)	46 XX	m-Bcr-Abl (type e1a2)	8.000/ul	Untreated
Sample 4	Very proliferative AML FAB4	46 XY, t (3;12) (q26;p13) [20]	No detectable mutations	269.000/ul	Two applications of hydroxyurea

## Data Availability

Data is contained within the article.
